# Das Reifegradmodell für den Öffentlichen Gesundheitsdienst – Ein Instrument zur Erfassung und Verbesserung des digitalen Reifegrades von deutschen Gesundheitsämtern

**DOI:** 10.1007/s00103-022-03643-7

**Published:** 2023-01-11

**Authors:** Torsten Eymann, Daniel Fürstenau, Martin Gersch, Anna Lina Kauffmann, Maria Neubauer, Doreen Schick, Nina Schlömer, Matthias Schulte-Althoff, Jeannette Stark, Laura von Welczeck

**Affiliations:** 1 Lehrstuhl für Wirtschaftsinformatik, Universität Bayreuth, Institutsteil Wirtschaftsinformatik des Fraunhofer FIT, Wittelsbacherring 10, 95444 Bayreuth, Deutschland; 2 Department of Business IT, IT University of Copenhagen, Kopenhagen, Dänemark; 3 Institut für Medizinische Informatik, Charité – Universitätsmedizin Berlin, Berlin, Deutschland; 4 Department Wirtschaftsinformatik, Freie Universität Berlin, Berlin, Deutschland; 5 Forschungsgruppe Digital Health, Technische Universität Dresden, Dresden, Deutschland

**Keywords:** Digitale Kompetenzen, Digitalisierung, E-Government, Digitale Reife, Öffentlicher Gesundheitsdienst, Digital competencies, Digitalization, E-Government, Digital maturity, Public health service

## Abstract

Die COVID-19-Krise verdeutlichte die Schlüsselrolle des Öffentlichen Gesundheitsdienstes (ÖGD) mit den rund 375 kommunalen Gesundheitsämtern in der Pandemiebekämpfung. Dabei stellte neben fehlenden personellen Ressourcen auch die unzureichende digitale Reife vieler Gesundheitsämter eine Hürde für die effektive und skalierbare Infektionsmeldung und Kontaktnachverfolgung dar. In diesem Artikel stellen wir das Reifegradmodell (RGM) für die Digitalisierung von Gesundheitsämtern vor, dessen Erarbeitung im Zeitraum Januar 2021 bis Februar 2022 stattfand und durch das Bundesministerium für Gesundheit gefördert wurde. Es findet seit Anfang 2022 Anwendung mit dem Ziel der Stärkung der Digitalisierung des ÖGD. Das RGM zielt darauf ab, Gesundheitsämter Schritt für Schritt anzuleiten, ihre digitale Reife zu erhöhen, um so für zukünftige Herausforderungen gerüstet zu sein. Entwickelt und evaluiert wurde das RGM anhand qualitativer Interviews mit Mitarbeitenden der Gesundheitsämter und weiteren Expert*innen des ÖGD sowie in Workshops und im Rahmen einer quantitativen Umfrage. Das RGM erlaubt die Messung der digitalen Reife in 8 Dimensionen, welche jeweils in 2–5 Subdimensionen untergliedert sind. Innerhalb der Subdimensionen erfolgt eine Einstufung auf 5 verschiedenen Reifegradstufen. Derzeit dient das RGM neben der Erfassung der digitalen Reife der einzelnen Gesundheitsämter auch als Management-Tool für die Planung von Digitalisierungsprojekten. Ziel ist es, auf Basis des RGM eine zielgerichtete Kommunikation zwischen den Gesundheitsämtern zu fördern, um Best Practices für die einzelnen Dimensionen auszutauschen.

## Abstract (en)

The COVID 19 crisis has highlighted the key role of the public health service (PHS), with its approximately 375 municipal health offices involved in the pandemic response. Here, in addition to a lack of human resources, the insufficient digital maturity of many public health departments posed a hurdle to effective and scalable infection reporting and contact tracing. In this article, we present the maturity model (MM) for the digitization of health offices, the development of which took place between January 2021 and February 2022 and was funded by the German Federal Ministry of Health. It has been applied since the beginning of 2022 with the aim of strengthening the digitization of the PHS. The MM aims to guide public health departments step by step to increase their digital maturity to be prepared for future challenges. The MM was developed and evaluated based on qualitative interviews with employees of public health departments and other experts in the public health sector as well as in workshops and with a quantitative survey. The MM allows the measurement of digital maturity in eight dimensions, each of which is subdivided into two to five subdimensions. Within the subdimensions a classification is made on five different maturity levels. Currently, in addition to recording the digital maturity of individual health departments, the MM also serves as a management tool for planning digitization projects. The aim is to use the MM as a basis for promoting targeted communication between the health departments to exchange best practices for the different dimensions.

## Einleitung

Die COVID-19-Pandemie stellte die Bedeutung des Öffentlichen Gesundheitsdienstes (ÖGD) in den Fokus [1]. Zahlreiche Berichte über Personalmangel [2, 3] und eine nicht ausreichende digitale Infrastruktur [4, 5] wurden von der Presse veröffentlicht. Zum Beispiel stellte die Kontaktnachverfolgung als wesentlicher Bestandteil der Eindämmung von COVID-19-Ausbrüchen [6, 7] angesichts der begrenzten personellen und IT-technischen Ressourcen eine Herausforderung dar. Während dieser Krise nutzten einige Gesundheitsämter selbsterstellte Excel-Tabellen zur Datenspeicherung und Faxgeräte zum Empfang von Laborberichten. Diese zeit- und personalintensiven Maßnahmen führten insbesondere in Zeiten der COVID-19-Pandemie zu Engpässen, so dass Mitarbeitende außergewöhnlicher Arbeitsbelastung ausgesetzt waren [8]. Die Digitalisierung von Gesundheitsämtern gilt deshalb spätestens seit der COVID-19-Pandemie als Voraussetzung, um Prozesse effizienter zu gestalten und Mitarbeitende zu entlasten. Im Rahmen des „Paktes für den Öffentlichen Gesundheitsdienst“ (ÖGD-Pakt) werden 4 Mrd. € für die personelle Aufstockung, Modernisierung und Vernetzung der deutschen Gesundheitsämter bereitgestellt, wobei der digitale Ausbau des ÖGD mit 800 Mio. € gefördert wird [9].

Der ÖGD spielt aber nicht nur eine zentrale Rolle bei der Pandemiebekämpfung, sondern auch bei Prävention sowie Gesundheitsschutz und -förderung der Bevölkerung. In Deutschland setzt sich der ÖGD aus den Einrichtungen der Gesundheitsverwaltung auf Bundesebene (z. B. Robert Koch-Institut – RKI), Länderebene (z. B. Ländergesundheitsministerien, Landesämter bzw. Landesinstitute für Gesundheit) und kommunaler Ebene (z. B. Gesundheitsämter) zusammen [10]. Auf kommunaler Ebene ist der ÖGD mit ca. 375 Gesundheitsämtern vertreten. Neben den akut anfallenden Aufgaben in der Bekämpfung der COVID-19-Pandemie, wie der Meldung von Infektionsfällen oder der Kontaktnachverfolgung, umfasst das Aufgabenspektrum der Gesundheitsämter dauerhaft relevante Aufgabenfelder, wie Beratungs- und Unterstützungsangebote für Individuen, Familien und Einrichtungen, den Kinder- und Jugendgesundheitsschutz, das amtsärztliche Bescheinigungswesen sowie Kontroll- und Überwachungsfunktionen, bspw. in der Krankenhaushygiene.

Der mit dem ÖGD-Pakt angestrebte digitale Ausbau der Gesundheitsämter unterliegt jedoch einigen strukturellen und technischen Rahmenbedingungen. So sind die Gesundheitsämter Teil der Landkreise und der kreisfreien Städte und damit in deren Organisation der IT-Infrastruktur und der digitalen Ausstattung eingebunden. Der überwiegende Anteil der Landkreise und kreisfreien Städte verfügt über eine IT-Abteilung, die die digitale Anbindung und Ausstattung der Gesundheitsämter verantwortet [11]. Für den Bereich der Infektionsprävention und -bekämpfung lässt sich hinsichtlich der Nutzung von Fachanwendungen ein heterogenes Bild zeichnen. Das RKI stellt den Gesundheitsämtern mit SurvNet@RKI das am häufigsten genutzte Tool zur Verfügung. Darüber hinaus werden zahlreiche andere Fachanwendungen wie OctoWare, ISGA, Äskullab 21 oder Mikropro genutzt [11]. Viele dieser Fachanwendungen decken ein Leistungsspektrum über den Infektionsschutz hinaus ab und können so vielseitig in den Gesundheitsämtern eingesetzt werden. Jedoch fehlen in kommerziellen Fachanwendungen häufig passende Schnittstellen, die z. B. notwendig sind, um eine nahtlose Kommunikation zu SORMAS zu ermöglichen. SORMAS ist eine Software, die deutschlandweit häufig zum Pandemiemanagement eingesetzt wird. Durch diese fehlende Interoperabilität im Bereich des Infektionsschutzes kommt es häufig noch zu manuellem Mehraufwand. Diese Heterogenität der technischen Landschaft und die unzureichende Interoperabilität erschweren zentral gesteuerte Digitalisierungsvorhaben. Weiterhin gibt es eine anhaltende Diskussion, dass die Gesundheitsämter in Deutschland auch aufgrund struktureller Gegebenheiten Schwierigkeiten bei der Digitalisierung haben [12, 13]. Die Digitalisierung findet in Deutschland aufgrund föderaler Strukturen oft in bundeslandspezifischen „Silos“ statt, was zu unterschiedlichen Fähigkeiten in den einzelnen Bundesländern führt [13]. Aufgrund dieser technischen und strukturellen Hemmnisse braucht es Unterstützung, um die in der Krise arbeitenden Gesundheitsämter schrittweise anzuleiten und mit einer einheitlichen Zielvorstellung zu digitalisieren. Für diese schrittweisen Vorhaben in Richtung eines gemeinsamen Ziels haben sich in der Vergangenheit Reifegradmodelle (RGM) bewährt. Diese Modelle beschreiben entlang definierter Reifegradstufen konkrete Entwicklungspfade, die insbesondere in komplexen technischen Projekten helfen, konkrete Maßnahmen abzuleiten, um die Digitalisierung sukzessive zu verbessern [14–16].

Bestehende Reifegradmodelle zeigen bislang Evidenzlücken sowohl in der Evaluation genutzter RGM als auch in der spezifischen Entwicklung eines RGM für den ÖGD. Um diese Evidenzlücken zu füllen, wurde das Projekt „Reifegradmodelle für die Unterstützung des Pakts für den öffentlichen Gesundheitsdienst“ (ReDiGe) ins Leben gerufen. Ziel dieses Projektes ist es, ein empiriebasiertes RGM für den ÖGD zu entwickeln und dieses unter Einbezug der Nutzenden zu evaluieren und zu optimieren. In diesem Beitrag wird das RGM als Messmodell für den digitalen Status quo deutscher Gesundheitsämter vorgestellt.

## Aktuelle Digitalisierungsstrategien im Gesundheitswesen

In der Literatur existiert bereits eine Vielzahl an RGM [17]. Im Kontext des Gesundheitswesens beschränken sich diese jedoch insbesondere auf den Bereich der Krankenhäuser [18], wobei besonders der DigitalRadar, der im Zuge des Krankenhauszukunftsgesetzes (§ 14a KHZG) entwickelt wurde, hervorsticht. Dabei handelt es sich um ein mehrdimensionales RGM, das dazu dient, den Digitalisierungsgrad der Krankenhäuser und die Verbesserungen der digitalen Reife der durch das KHZG finanziell geförderten Krankenhäuser zu evaluieren. Anhand dessen schätzen sich die Krankenhäuser im Abstand von 2 Jahren (06/2021 und 07/2023) selbst ein [19]. Damit weist der DigitalRadar einige Ähnlichkeiten zum Reifegradmodell für den ÖGD auf, richtet sich mit den Krankenhäusern aber an grundlegend andere Akteure mit anderen Digitalisierungsdimensionen.

Während der DigitalRadar ein recht junges Beispiel für die Erfassung der digitalen Reife im Gesundheitswesen ist, wird durch den *IT-Report Gesundheitswesen *der Hochschule Osnabrück seit 2002 regelmäßig der Stand der Digitalisierung in Krankenhäusern in Deutschland erhoben [20]. Eine vergleichende Analyse untersuchte diesen IT-Report und 41 andere Reifegradmodelle für Krankenhäuser und andere Einrichtungen der Gesundheitsversorgung und kritisiert die mangelnde wissenschaftliche Fundierung in der Modellentwicklung, einen einseitigen Fokus auf die technische Umsetzung in der Organisation, die fehlenden Möglichkeiten zur Ableitung konkreter Handlungsempfehlungen [21] sowie die bestehende Trennung zwischen dem Erreichen einer digitalen Reife und der Nutzenstiftung durch Digitalisierung [22].

Durch den Digital-Health-Index der Bertelsmann-Stiftung wird auf einer Makroebene der Digitalisierungsstand des Gesundheitswesens einzelner Länder in 3 Dimensionen erfasst und in den internationalen Vergleich gestellt. Im Gegensatz zu Reifegradmodellen werden hierbei die Länder a posteriori je nach erreichtem Index in Gruppen eingeteilt, anstatt vorher Reifegradstufen festzulegen. Allerdings werden auch hier nicht die Akteure des ÖGD, sondern vielmehr die klassischen Träger der medizinischen Versorgung fokussiert [23].

Speziell für den in der Krise agierenden ÖGD gibt es kaum bestehende Ansätze und lediglich geringfügige Forschungs- und Entwicklungsinitiativen. Das in diesem Bericht vorgestellte RGM für den ÖGD soll diese Lücke schließen. Es entstand in enger Zusammenarbeit von Expert*innen der Digitalisierung des öffentlichen Sektors, Praktiker*innen aus dem ÖGD, Vertreter*innen des Bundesministeriums für Gesundheit (BMG) und zahlreichen Berater*innen aus Wissenschaft und Wirtschaft.

## Modellentwicklung

**Figure 1:**
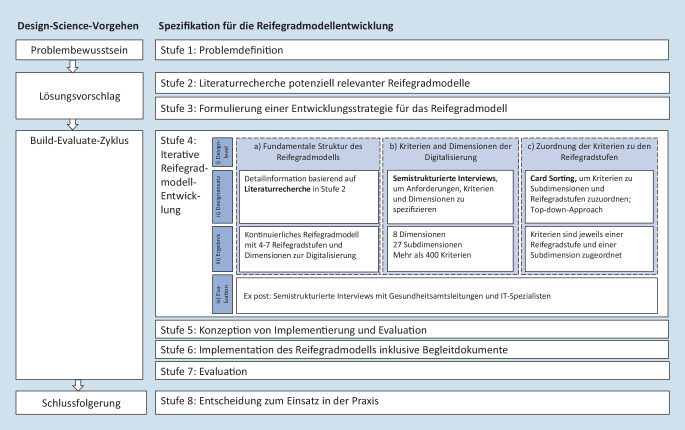

Die Entwicklung des RGM für die Digitalisierung der Gesundheitsämter erfolgte im Zeitraum Januar 2021 bis Februar 2022 im Rahmen eines gestaltungsorientierten Vorgehens (Design Science). Diese Methode beinhaltet 4 Schritte: Problembewusstsein, Lösungsvorschlag, Build-Evaluate-Zyklus und Schlussfolgerung [24, 25]. Um die Methode bestmöglich im Kontext der Entwicklung eines RGM für den ÖGD umzusetzen, orientierte sich das Forschungsteam am 8‑stufigen Modell zur Entwicklung von RGM [16], das im folgenden Text vorgestellt wird (Abb. 1).

Die erste Stufe zur Entwicklung von RGM, „Problemdefinition“, wurde bereits in der Einleitung beschrieben. Für Stufe 2 wurde die Literatur zur Reifegradmodellforschung in Gesundheitsorganisationen recherchiert und analysiert. Dabei wurden auch die Bereiche Wissensmanagement und Softwareentwicklung betrachtet [26–29]. Diese Recherche zeigte, dass zu dieser Zeit kein passendes RGM für die Digitalisierung von Gesundheitsämtern existierte. Dennoch konnte bestehendes Wissen aus der Literaturrecherche in Stufe 3, der „Formulierung einer Entwicklungsstrategie für das RGM“, verwendet werden. Auf Basis der Resultate der Literaturrecherche fiel die Entscheidung auf einen Top-down-Ansatz zur Erstellung des RGM. Das daraus resultierende RGM soll es Gesundheitsämtern erlauben, ihren digitalen Reifegrad zu evaluieren [30] und sich für einzelne Bereiche zu entscheiden, in denen sie sich mit Hilfe von Digitalisierungsprojekten verbessern wollen. Ziel des Reifegradmodells ist es dann, konkrete Schritte darzustellen, wie die digitale Reife in diesen Bereichen verbessert werden kann. In Stufe 4 (iterative Reifegradmodellentwicklung) wurde das RGM entsprechend der folgenden 3 Gestaltungsebenen entwickelt: a) die fundamentale Struktur des RGM, b) die Kriterien und Dimensionen der Digitalisierung und c) die Zuweisung der Kriterien zu den Reifegradstufen und Subdimensionen (siehe a–c in Abb. 1). Je Gestaltungsebene wurden der Gestaltungsansatz, das Ergebnis und die Bewertung festgelegt (ii–iv). Die grundlegende Struktur des RGM basiert dabei auf den Ergebnissen der strukturierten Literaturrecherche aus Stufe 2 und beinhaltete 5 kontinuierliche Reifegradstufen. Anschließend konnte das RGM auf Basis von halbstrukturierten Interviews mit 43 Expert*innen aus dem ÖGD weiter spezifiziert werden, indem Kriterien und Dimensionen abgeleitet wurden (b, c). Die Interviews hatten insbesondere das Ziel, den Digitalisierungskontext zu erfassen, den Umfang des RGM zu definieren, einen erreichbaren Reifegrad abzuleiten sowie Kriterien, Dimensionen und Subdimensionen der Digitalisierung in Gesundheitsämtern zu bestimmen.

Um die Kriterien den Reifegraden (c) zuzuordnen, wurden zunächst alle Kriterien in Dimensionen und Subdimensionen geclustert. Daraufhin ordneten die am Projekt ReDiGe Beteiligten mit Hilfe der Card-Sorting-Methode die Kriterien jeweils einer Subdimension und einer der 5 Reifegradstufen innerhalb dieser zu. Auf diese Weise entstand ein RGM mit 27 Subdimensionen und 8 übergeordneten Dimensionen, in denen die einzelnen Kriterien 5 Reifegraden zugeordnet sind. Um das resultierende RGM zu evaluieren, führte das ReDiGe-Projekt sowohl eine Ex-ante- als auch eine Ex-post-Evaluation des RGM durch [31]. Im Sinne einer Ex-ante-Evaluation wurde der RGM-Prototyp in einer weiteren Interviewrunde mit 18 Gesundheitsamtsleitungen und IT-Spezialist*innen evaluiert (iv). Den Interviewteilnehmenden wurde dabei das RGM vorgelegt. Sie wurden gebeten, Kriterien bei Bedarf neu zuzuordnen oder die Beschreibung der Kriterien zu ändern, falls diese ihrer Meinung nach nicht passten, oder fehlende Kriterien zu ergänzen. Die Ergebnisse dieser Interviewrunde wurden dann im ReDiGe-Team diskutiert und gegebenenfalls in Form von Änderungen am RGM übernommen. Bei den Interviews und Workshops wurde stets auf die Inkludierung eines möglichst breiten Spektrums an Expert*innen und Mitarbeitenden des ÖGD geachtet, damit das RGM später bestmöglich anwendbar, nutzerfreundlich und umfassend ist. So konnten mit Hilfe der verschiedenen Perspektiven die Kriterien und (Sub)Dimensionen über die vielen Iterationen bei der Entwicklung und Evaluierung des RGM weiter verfeinert und entwickelt werden.

Für die Ex-post-Evaluation des RGM wurde das RGM der Zielgruppe der deutschen Gesundheitsämter und anderen Stakeholdern des ÖGD als verwendbares Bewertungsinstrument (als Excel-Datei) zusammen mit unterstützenden Dokumenten (z. B. Benutzerleitfaden, Handlungsempfehlungen für die Verbesserung im RGM) über eine deutschlandweite Mailingliste zur Verfügung gestellt (Stufe 5–6). Für eine abschließende Bewertung (Stufe 7) und um die Herausforderung der Kontextabhängigkeit zu überwinden, wurde eine Umfrage durchgeführt, die sich an alle deutschen Gesundheitsämter richtete. Damit sollte das RGM auf Vollständigkeit, Konsistenz und Problempassung geprüft werden [16]. Insgesamt gingen 34 Antworten auf die Umfrage ein. Weiterhin wurde eine erste empirische Evaluation des RGM mit 15 Personen aus insgesamt 12 Gesundheitsämtern durchgeführt [31]. Die Teilnehmenden wurden dabei angewiesen, das RGM auszufüllen und dabei ihre Gedanken laut zu äußern. Durch diese Methode des lauten Denkens (engl. „think aloud“) haben die Forschenden die Teilnehmenden im Umgang mit dem RGM beobachtet und gezielte Rückfragen, z. B. zum Verständnis oder zur Vollständigkeit des RGM, gestellt. Insgesamt zielte diese erste, kleinere Evaluierung darauf ab, festzustellen, ob das RGM zum anfangs festgelegten Problem passt, einen Nutzen schafft und das vorhandene Wissen über die Digitalisierung von Gesundheitsämtern erweitert. Auf der Grundlage der Evaluierungsergebnisse beschloss das ReDiGe-Team, das RGM zur Verfügung zu stellen und in der Praxis einzusetzen (Stufe 8).

## Einsatz des Reifegradmodells (RGM) für den Öffentlichen Gesundheitsdienst (ÖGD)

Auf Basis der beschriebenen Prozessstufen wurde das RGM für den ÖGD erarbeitet. Im Kern besteht es aus einem umfangreichen Kriterienkatalog mit mehr als 200 Kriterien, deren Beantwortung die digitale Reife von Gesundheitsämtern evaluiert. Jedes einzelne Kriterium erfragt, inwieweit ein bestimmter Zustand im Gesundheitsamt vorliegt. Dazu kann aus den 3 Antwortmöglichkeiten „trifft zu“, „in Umsetzung“ oder „trifft nicht zu“ ausgewählt werden.

Das Reifegradmodell und somit auch der Fragenkatalog sind in 8 Dimensionen unterteilt, entlang derer die digitale Reife gemessen wird: Digitalisierungsstrategie, Mitarbeitende, Prozessdigitalisierung, IT-Sicherheit, IT-Bereitstellung, Bürger*innenzentrierung, Zusammenarbeit und Software, Daten und Interoperabilität (Tab. 1). Jede dieser Dimensionen besteht aus weiteren Subdimensionen, so dass eine feingranulare Einstufung in den einzelnen Digitalisierungsaspekten sowie das Erkennen spezifischer digitaler Fähigkeiten ermöglicht wird. Ein Teil des Reifegradmodells adressiert die für die Gesundheitsämter typische Einbettung in die darüberliegenden Behörden, wie zum Beispiel Landratsämter, die in der Regel die Organisation der IT verantworten (Dimensionen „IT-Bereitstellung“ und „IT-Sicherheit“). Weiterhin adressiert das Reifegradmodell auch spezifische Aspekte von Gesundheitsämtern, wie zum Beispiel die Aufbereitung ihrer Dienste für Bürger*innen (Dimension „Bürger*innenzentrierung“), oder die Zusammenarbeit mit den zahlreichen Stakeholdern von Gesundheitsämtern, wie z. B. Ärzt*innen, Polizei, Notar*innen, Krankenhäusern und Rehakliniken (Dimension „Zusammenarbeit“).

**Table 1:** 

Dimension		Beschreibung (kursiv hervorgehoben sind die Namen der Subdimensionen)
1	Digitalisierungsstrategie	Diese Dimension misst den Reifegrad der übergeordneten Digitalisierungs-Roadmap des Gesundheitsamtes anhand ihrer *Definition, Kommunikation und Umsetzung* sowie der klaren Zuteilung von *Verantwortlichkeiten* und eines *Digitalisierungsbudgets*
2	Mitarbeitende	Zentral für die erfolgreiche Umsetzung der Digitalisierungsstrategie ist das Mitwirken der Mitarbeitenden, welches anhand von digitalisierungsbezogener *Sensibilisierung, Partizipation* und der Organisation von* Schulungen* gemessen wird
3	Prozessdigitalisierung	Um die für eine erfolgreiche Digitalisierung notwendige Prozessorientierung ganzheitlich zu denken, ist zunächst die *Dokumentation* aktueller Prozesse und ihrer IT-Unterstützung notwendig, auch über fachdienst*übergreifende Prozesse* hinweg. Nicht nur die Erfassung, sondern auch die kontinuierliche *Evaluation* dieser Prozesse ist hierbei zentral
4	IT-Sicherheit	Diese Dimension erfasst, inwieweit das Gesundheitsamt entsprechend eines *IT-Sicherheitsmanagements* einen souveränen *Umgang mit IT-Sicherheitsrisiken und Angriffen* gefunden sowie ein den Anforderungen entsprechendes *Identitäts- und Zugangsmanagement* implementiert hat
5	IT-Bereitstellung	Als hardwaretechnische Grundlage der Digitalisierung misst diese Dimension die *Ausstattung des IT-Arbeitsplatzes* sowie die vorausschauende *Organisation der IT-Beschaffung* und den *Bezug von IT-Infrastruktur*. Um die Effizienz und Effektivität von IT-Services sicherzustellen, erfasst die Dimension auch den Grad von *IT-Service-Prozessen*
6	Bürger*innenzentrierung	Insbesondere in der Pandemie hat sich die Bedeutung der Orientierung an den Bedürfnissen der Bürger*innen gezeigt. Hierzu zählt sowohl die digitale und nichtdigitale *Interaktion* mit Bürger*innen sowie die bürger*innenseitige Möglichkeit, *Präferenzen* bei der Wahl der Kommunikationswege zu setzen
7	Zusammenarbeit	Diese Dimension umfasst sowohl die abteilungsübergreifende *Zusammenarbeit innerhalb des Gesundheitsamtes* als auch die organisationsübergreifende *Zusammenarbeit zwischen* *verschiedenen Gesundheitsämtern und mit den Landesstellen* sowie mit externen Stakeholder*innen wie Krankenhäusern oder Gerichten
8	Software, Daten und Interoperabilität	Den softwareseitigen Kern der Digitalisierung in den Gesundheitsämtern umfassend, misst diese Dimension den digitalen Grad der *Fachanwendungen* und deren *technische Interoperabilität* sowie vorhandene Möglichkeiten zur *Datenanalyse und Berichterstattung*. Zentral sind hier neben dem *Datenschutz* auch die *Anforderungen und Dokumentationen des Fehlermanagements*

Jede Subdimension unterteilt sich in 5 Reifegrade. Grad 0 steht dabei für einen geringen digitalen Reifegrad, während der Grad 4 eine hohe digitale Reife beschreibt. Um einen höheren Reifegrad einer Dimension oder Subdimension zu erreichen, müssen jeweils mindestens 80 % der Kriterien einer Dimension für diesen Grad erfüllt sein sowie die vorhergehenden Reifegrade erreicht sein. Dies ist immer dann der Fall, wenn in einer Stufe 80 % der Kriterien mit „trifft zu“ beantwortet wurden. Das RGM ist derzeit über die Website „Gesundheitsamt 2025“^1^ des BMG in der jeweils aktuellen Version als Excel-Variante sowie als Web-Tool zugänglich. Darüber hinaus werden auf dieser Seite Begleitdokumente zur Verfügung gestellt, die die Nutzung des RGM erleichtern und seine korrekte Anwendung sicherstellen sollen.

## Ausblick

Das empiriebasiert entwickelte RGM erfüllt verschiedene Funktionen für die Digitalisierung von Gesundheitsämtern. Neben der Ermittlung des Ist-Zustandes der digitalen Reife in den 8 Dimensionen kann mit Hilfe des RGM ein digitaler Soll-Zustand bestimmt werden. Um diesen Soll-Zustand zu erreichen, können Digitalisierungsprojekte aus den einzelnen Kriterien abgeleitet werden und als kurzfristige, mittelfristige und langfristige Digitalisierungsprojekte priorisiert werden. Zur Erarbeitung der digitalen Fähigkeiten wurden im Projekt ReDiGe weiterhin Handlungsempfehlungen erarbeitet. Diese fassen erste Best Practices innerhalb der Dimensionen und Subdimensionen zusammen und helfen so bei der Definition und Ausarbeitung von Digitalisierungsprojekten. Insofern kann das RGM den Gesundheitsämtern dabei helfen, eine klare Digitalisierungsvision zu formulieren und die Schritte auf dem Weg zu dieser Vision transparent und realisierbar darzustellen und umzusetzen [14–16].

Das RGM bildet die Grundlage für eine bundesweite, regelmäßige Evaluation der digitalen Reife der Gesundheitsämter in einem Anschlussprojekt (EvalDiGe). Zudem bildet das RGM auch den Referenzrahmen für den ersten Förderaufruf 2022 im Rahmen des ÖGD-Paktes, bei dem Digitalisierungsvorhaben von Einrichtungen des ÖGD auf kommunaler oder Landesebene gefördert werden [32]. Voraussetzung und Ausgangspunkt dieser Förderung ist die Einstufung in das RGM. Anhand der Einordnung ins RGM wird anschließend der initiale digitale Reifegrad auf Landes- und Bundesebene ermittelt. Jährliche Folgemessungen bis 2025 sollen es ermöglichen, den Anstieg der digitalen Reife der Gesundheitsämter und weiterer ÖGD-Institutionen auf Basis der Maßnahmen aus dem RGM sowie die Effizienz der Förderung dieser zu evaluieren. Bei der Interpretation der Evaluationsergebnisse zum digitalen Reifegrad des ÖGD ist zu beachten, dass die Reifegradmessung der Limitation der Subjektivität unterliegt, da die Gesundheitsämter eine Selbsteinstufung ohne äußere Kontrolle vornehmen. Um diese Limitation zu minimieren, wurden zahlreiche Begleitmaterialien und Workshops zur Sensibilisierung für den richtigen Umgang und ein konsistentes Verständnis zum RGM durch das EvalDiGe-Projekt durchgeführt. Weiterhin ist es Ziel des Projektes EvalDiGe, das RGM auf Basis der Ergebnisse der Einordnungen und gezielter Interviews weiter zu verbessern und möglichst anwendungsfreundlich zu gestalten. Diese Verbesserung zielt insbesondere darauf ab, die Kriterien verständlicher zu formulieren und an aktuelle Rahmenbedingungen und Entwicklungen anzupassen. Ein weiteres Ziel des Projektes EvalDiGe ist es, das RGM als „lebendes“ RGM auszubauen, so dass ÖGD-Teilnehmende Erfahrungen zu bereits erfolgten Maßnahmen gezielt für die Kriterien formulieren oder bereits hinterlegte Erfahrungen kommentieren und ergänzen können. Das „lebende“ RGM soll damit einen Erfahrungsaustausch zwischen den Gesundheitsämtern und weiteren ÖGD-Teilnehmern ermöglichen und den Prozess der Digitalisierung weitergehend erleichtern.

## Fazit

Das ab 2021 neu entwickelte Reifegradmodell (RGM) zur Erfassung der digitalen Reife des Öffentlichen Gesundheitsdienstes (ÖGD) wurde unter Einbezug von Praxis und Forschung ausgearbeitet und ist somit möglichst allumfassend und praxisnah gestaltet. Es soll mit seinen 8 Dimensionen nun als Unterstützung dienen, die Digitalisierung im ÖGD nachhaltig voranzutreiben und als eine Art Schablone und Wegweiser zur Digitalisierung unter Berücksichtigung der besonderen Strukturen und Anforderungen des ÖGD auch über die Projektlaufzeit hinaus dienen. Dabei soll auch die Weiterentwicklung des RGM im Zuge der Evaluation helfen, bei der das RGM letztendlich auch als „lebendes“ RGM ausgebaut und langfristig als Austauschmedium zur Verfügung gestellt werden soll.

## Footnotes

1 https://gesundheitsamt-2025.de/digitalisierung/reifegradmodell; zuletzt abgerufen am 06.12.2022.
